# Bromide of Potassium

**Published:** 1872-05

**Authors:** K. P. Moore

**Affiliations:** Knoxville, Georgia


### BROMIDE OF POTASSIUM.

BY K. P. MOORE, M.D., KNOXVILLE, GEORGIA.

[Read Before the Georgia Medical Association, at its Annual Meeting, in Columbus, 1872.]

The various virtues of bromide of potassium have, of late years, attracted the attention of the medical profession in every direction. Many pages of the medical periodicals of our day shine with reports of excellent results, in the treatment of a variety of diseases, from its employment; and yet we think there is room. My experience testifies to its use as an alterative, a nervine, a cardiac sedative—or, rather, arterial equalizer—and a powerful sedative to the brain and spinal axis. My remarks, however, will be confined more particularly to the three latter virtues, because better and more efficacious alteratives are in use; and where only a general alterative is indicated, I would not urge the use of the bromide before some of the more powerful remedies now used.

Passing, then, over its use as a general alterative, I come to notice its value as a nervine; and for the sake of brevity, I will notice its use both as a nervine and brain sedative together, for really the one may be the result of the other. I do not remember ever to have seen any account of its employment in the treatment of very young children, where there was an indication for a nervine or sedative to the brain—such, for instance, as a tendency to the development of brain symptoms from almost any cause whatever. The idea of its use here is original with me, and presents, so far as I have been able to ascertain, a new employment of it; and hence, I invite attention more particularly to this point than any other in this paper.

We are sometimes called upon to treat convulsions in children, and are at a loss to ascertain the cause, from the incapacity of the little sufferers to tell us of their pains; nor does the previous history throw any light upon the case. To explain myself more fully, I take from my case-book the following:

April 11th, 1871, was called to see a girl-child of S. R. H., five weeks old. Found her very weak—scarcely able to make an audible noise in attempting to cry; a living skeleton; was constantly having convulsions. I learned from the family that she had been having convulsions since she was two weeks old; the family could not give any reason why she was thus affected, nor could I determine upon a rational reason. I was told that a medical friend of mine—a member of this Association—had seen the case a week before, and declined doing anything, except, perhaps, giving a little hydrarg. cum creta—stating that for a child so young and so weak, what the parents could not do by simple teas and hygienic measures, he could not do with medicine; doubtless, in general, a wise conclusion. This friend of mine, I felt, and so expressed myself to the family, was my superior both in age and experience. I had always regarded him as a skillful physician, and hence felt a delicacy in making a prescription, and especially since the little babe seemed just "waiting at the river." Yet, I wished to try the bromide of potassium, as I was then making notes of its use with quite young children; hence, made the following:

℞—Potass. brom., grs. xvj; extract hyosciam., grs. ij; aqua ℥ ij. M.—Give a tea-spoonful every four hours.

Feeling almost certain that the babe would die very soon, I left no appointment for a subsequent visit; told the parents if patient improved, to imform me. Three days later, having to pass, I called, and was greatly surprised and gratified to learn that from the very beginning of the medicine, my little patient began to improve. Continued the same prescription; and a few weeks later, I saw my patient—a bright, living monument, through Almighty God, I doubt not, to the virtues of bromide of potassium. Other cases have come under my care, with equally as marked benefit.

To return: As before intimated, in a tendency to convulsions, from almost any cause—whether from reflex nervous impression, or from inflammatory fever—the bromide is not contra-indicated; and by it, I have no doubt, we can frequently control and hold in abeyance the convulsions—at least, stay the paroxysms until we can remove, or allow Nature herself to remove, the disturbing cause.

The various other remedies—preparations of opium, alcoholic tinctures, etc.—ordinarily used as nervines, antispasmodics, etc., are contraindicated in cases where there seems to be an already-existing predisposition to the development of brain symptoms, from their tendency to increase that development; while, on the other hand, the bromide, from its action as a powerful sedative to the brain and spinal axis, and also as a cardiac sedative, is, in almost all such cases, eminently indicated—in fact, *the very thing* indicated.

Not only is it useful in children, but in all cases where there is nervous excitement and spasmodic nervous action. I need scarcely say that nothing is more gratifying than the results obtained from its use in hysterical and epileptico-hysterical seizures; and I am not certain but that epilepsy, in young and recent cases, may be, I am almost tempted to say, cured by it. I have now on record, in my case-book, two cases of children, under five years of age, whom I treated with it more than two years ago, who had what I diagnosed as confirmed epileptic fits; and by a long-continued perseverance in the remedy, they ceased to have them, and no return in two years. Of course, other measures were not neglected, where the indications called for them.

Experience is said to be the best teacher; and in this connection, please allow me to go again to my case-book, as I can, I think, better fix the mind upon the information designed to be conveyed by this paper, by reference to actual experience than by speculative ideas.

During the month of December, four of J. J. C——'s family were stricken down with what I diagnosed as severe influenza, with, perhaps, more or less meningeal disturbance. In one case, the youngest, eighteen months old, there were paroxysms of convulsions, followed by coma; in one of the others, fifteen years old, light convulsions, with raving delirium; and in all, great restlessness and complaining of pain in the head, with high fever.

Without going into detail as to the treatment pursued in these cases, and following up my ideas on bromide of potassium, I thought, from my experience with the drug, and its known action, that it was certainly indicated; hence, I gave them heavy doses of it, with the gratifying results of keeping my patients quiet, or nearly so—of nearly controlling the convulsions entirely, and of keeping under control, or at least equalizing, the circulation.

These cases, notwithstanding their severity, and notwithstanding they were regarded by myself as dangerous, all recovered. Now, how far their recovery was attributable to the bromide, I will not pretend to say, or whether it had anything at all to do with their recovery, I can not tell; I simply mention the cases here to call attention to its action in the way I then designed—viz, to control excessive nervous and arterial excitement—which it did, much to my satisfaction.

I will give a synopsis of one other case.

Was called, January 29th, 1872, to Col. S. L. P——, about fifty years of age—a man of very nervous temperament, short, thick, chuffy make—a high liver, and of sedentary habit. He complained of pain in the head, peculiar uneasy sensation in side of the face, a want of proper control of the tongue and ideas; very nervous, excessively restless, and yet a great tendency to stupor. Diagnosis—More or less congestion of the brain, threatening apoplexy.

My treatment consisted simply of giving a brisk cathartic of calomel, and placing my patient on bromide potassium grs. xx. every four hours. Remained all night. Patient soon became quiet; partial paralysis of one side of face, tongue and upper extremity supervened. Persevered in the same prescription, using stimulating applications to the extremities and counter-irritants along the spine. The congestion gradually subsided, and recovery followed.

By February 6th, I discharged the patient, putting him on bromide of potassium and sarsaparilla three times per day for thirty days. In a few days, he left off the medicine, when he again became nervous, and somewhat threatened with brain trouble. I was again sent for. All I did was to resume the bromide, with the same gratifying results. Scarcely a doubt rests upon my mind but that the bromide kept him from an attack of apoplexy, which seemed so fearfully to threaten in the beginning.

I take occasion just here to thank Dr. Mathews, of Fort-Valley, for kindly seeing this patient with me, and his confirmation of my treatment.

Before closing, I will call attention to the experience of Dr. J. G. Thomas (and, as he states, of others of his medical friends in Savannah), contained in the *Medical Record*, January 15th, 1872, page 524, in the use of this drug in the treatment of dropsies. Also to the opinions of Dr. Benjamin S. Purse, of Savannah, Georgia (*Medical Record*, March 1st, 1872, page 28), on the many virtues of this drug, but especially its use in the treatment of yellow fever. I take the following from Dr. Purse's article:—"Bromide of potassium is also specific in albuminuria. It almost uniformly produces absorption of dropsical effusions, irrespective of their cause, and without sensibly increasing the secretions, unless they have been suppressed. It relieves congestions in every part of the body, particularly of the brain. It is *the remedy* in congestive and nervous headaches. It is a powerful sedative to the brain and spinal axis. It produces a sedative action on mucous membranes. It will relieve suppression of the urine."

While my experience and observation does not sustain the encomiums bestowed upon the remedy in the article quoted, nor while, indeed, they scarcely seem sustained by reason or by our knowledge of the power of medicine, yet it may serve to direct our attention to a medicine of high value, and one well worthy of further investigation.

